# Laparoendoscopic Single-Site Surgery Versus Conventional Laparoscopic Surgery in Ovarian Cystectomy: A Meta-Analysis

**DOI:** 10.3390/jcm14248739

**Published:** 2025-12-10

**Authors:** Greg J. Marchand, Ahmed T. Abdelsattar, Daniela Gonzalez Herrera, Mckenna Robinson, Emily Kline, Sarah Mera, Michelle Koshaba, Nidhi Pulicherla, Ali Azadi

**Affiliations:** 1 Marchand Institute for Minimally Invasive Surgery, 10238 E. Hampton, Ste. 212, Mesa, AZ 85209, USA; 2 Faculty of Medicine, Fayoum University, Fayoum 63514, Egypt; 3 College of Medicine, University of Arizona, Phoenix, AZ 85004, USA; 4 School of Medicine, Creighton University, Phoenix, AZ 85012, USA

**Keywords:** ovarian cystectomy, laparoendoscopic single-site surgery, conventional laparoscopic surgery, minimally invasive surgery, meta-analysis

## Abstract

**Highlights:**

**What are the main finding?**

- Laparoendoscopic single-site (LESS) ovarian cystectomy significantly reduces hospital stay by an average of 0.26 days compared to conventional multi-port laparoscopy.
- LESS cystectomy increases operative time by an average of 9.42 min but shows no significant differences in postoperative pain, blood loss, or complication rates versus conventional laparoscopy.

**What is the implication of the main finding?**

- LESS surgery provides a meaningful recovery advantage through shorter hospitalization with comparable safety and postoperative pain profiles.
- Despite longer operative times, LESS ovarian cystectomy is confirmed as a reliable and cosmetically superior alternative to conventional laparoscopy for benign ovarian cysts.

**Abstract:**

**Background/Objectives**: To systematically compare the effectiveness and safety of laparoendoscopic single-site surgery (LESS) versus conventional multi-port laparoscopic surgery (CLS) for ovarian cystectomy in women with benign ovarian cysts, focusing on operative outcomes, postoperative pain, and recovery parameters. **Methods**: A comprehensive search was conducted in PubMed, Cochrane Library, Web of Science, ClinicalTrials.gov, and SCOPUS from inception to 1 June 2024. Randomized controlled trials and observational studies directly comparing LESS with CLS for benign ovarian cystectomy (excluding adnexectomy) were included. Primary outcomes were operative time, blood loss, hospital stay, postoperative pain, and complications. Data were pooled using mean differences (MD) or risk ratios with 95% confidence intervals in fixed- or random-effects models according to heterogeneity. **Results**: Nine studies (1 randomized trial, 8 observational; total n = 1368 patients: 702 LESS, 666 CLS) were included. LESS was associated with longer operative time (MD 9.42 min, 95% CI 3.47–15.37, *p* = 0.002) but shorter hospital stay (MD −0.26 days, 95% CI −0.39 to −0.14, *p* < 0.001). No significant differences were found in blood loss, postoperative analgesic requirements, hemoglobin drop, or complication rates. Postoperative pain scores at 6 and 24 h were similar, although adjusted analysis suggested marginally lower pain at 24 h with LESS (MD −0.20, *p* = 0.05). **Conclusions**: Laparoendoscopic single-site ovarian cystectomy is a safe and reliable alternative to conventional multi-port laparoscopy, offering the advantage of shorter hospital stay despite slightly longer operative time, with equivalent safety profile and postoperative pain. LESS expands minimally invasive options in benign gynecologic surgery 1.1 s.

## 1. Introduction

Non-malignant ovarian growths are frequent conditions, mainly in females of reproductive age, with approximately 7% of detected growths causing symptoms [1]. Though many minor growths are benign or without symptoms and require no intervention, some necessitate operation due to twisting, growth, or rupture [1]. Multi-port laparoscopic excision has become established as a typical approach for managing these growths, providing several advantages over open surgery, including superior view during procedure, reduced cut dimensions, decreased fluid loss, lowered postoperative issues, briefer inpatient periods, and quicker complete healing [2,3].

Single-port endoscopic (LESS) has been created and implemented in practice as a form of low-impact operation. Gains from utilizing LESS include superior cosmetic outcomes, decreased postoperative issues, and briefer inpatient periods relative to multi-port laparoscopic (CLS) [4,5].

Of late, single-port operations have risen owing to progress in operational tools, cosmetic preferences, and the trend toward reduced intrusion techniques. LESS utilizes distinctive umbilical attributes, such as concealing bigger cuts without evident marks, being nearest to internal space (absence of muscle layer under the umbilicus), and diminished marks after operation because of the umbilical tissue nature [5,6]. Hence, umbilical access usually experiences minimal strain during wound sealing and forms less scar tissue compared to alternative abdominal entry sites [7].

In gynecologic operations, multiple investigations have probed the practicality and dependability of LESS, encompassing its application in gynecologic oncology, ectopic pregnancy management, and accessory organ operations, as well as uterine excisions. Still, few have directly contrasted LESS to multi-port laparoscopy to ascertain if it offers the benefit of lowering postoperative discomfort [8,9,10,11,12,13].

In females before menopause with non-malignant accessory growths, growth excision is commonly performed to maintain reproductive ability. However, the single-port method can be technically demanding, including the need for proficiency in advanced laparoscopic skills, such as achieving pull and counter-pull for growth excision, plus controlling bleeding from various origins with imaging and tools from a single access. Our objective with this meta-analysis is to probe the disparities in operational outcomes, postoperative discomfort, and the recuperation process, described as postoperative discomfort and recuperation indicators shown in inpatient time between single-port and multi-port for excision in reproductive-age females with non-malignant accessory growths, to evaluate and appraise the performance of this method.

## 2. Methods

We carried out our investigation according to the most recent PRISMA directives and suggestions [14]. The PRISMA list can be located in Additional Table S1. The investigation was pre-enrolled in the PROSPERO registry for aggregated reviews on 5 July 2024. The enrollment ID is CRD42024566126 [15].

Query Approach and Sources

We formulated a query plan by merging these terms: (“Laparoscopic operation” OR “laparoscopic procedure” OR “low-impact” AND “single-port endoscopic procedure” OR “LESS” AND “multi-port laparoscopic method” OR “CLS” AND “ovarian growth excision” AND “non-malignant ovarian conditions”). We explored six repositories: PubMed (Medline), Cochrane Collection, Web of Science, ClinicalTrials.gov, and SCOPUS. We covered search scores spanning from each repository’s origin to 1 June 2024.

Report Choice

The filtering phases were handled by two separate reviewers. Conflicts were resolved by reviewer agreement. Initially, they performed title and summary filtering, then complete text filtering. A third reviewer was designated to address any conflict between the pair, but no conflicts occurred. We chose the reports based on the following standards:

- Subjects: Females with non-malignant ovarian growths undergoing excision without tube removal, ovary removal, or combined.
- Approach: Single-port endoscopic procedure (LESS).
- Comparison: Multi-port laparoscopic method (CLS).
- Parameters: Indicators of operational outcomes (e.g., duration, fluid loss), postoperative discomfort, issues, and recuperation indicators (e.g., inpatient time).
- Format: We incorporated randomized trials (RCTs) and non-randomized investigations.

### 2.1. Quality Assessment

To gauge the quality of the chosen reports, we applied the Cochrane risk appraisal instrument for RCTs. Moreover, we applied the NHLBI quality assessment instruments to gauge the quality of the non-randomized investigations. Each investigation’s risk was grouped as low, high, or uncertain [16].

### 2.2. Data Collection

Retrieval was performed in three groups:

− Demographic Details: This encompassed basic traits of the subjects, such as age and weight measures.
− Parameters: Information on duration, fluid loss, postoperative discomfort (gauged by the Visual Scale, VAS), issue rates, and inpatient length.
− Quality Appraisal Information: Details from the quality assessment of each investigation.

Microsoft Excel was employed to arrange and handle the information gathering process. The demographic and basic details for all chosen investigations are in Table 1 and Table 2.

### 2.3. Statistical Analysis

Combined examinations and initial summary diagrams were executed using Review Manager 5.4.1 (The Cochrane Group). OpenMeta[Analyst] (Version Yosemite (10.10)) [23] was utilized solely for extra subgroup examinations needing variable-effects DerSimonian–Laird calculations when Review Manager results were inadequate for display. Continuous parameters were merged using mean difference (MD) with 95% confidence intervals; binary parameters used risk ratio (RR) with 95% CI. Fixed-effects approaches were used when variability was low (I^2^ ≤ 50% and *p* ≥ 0.1); otherwise, variable-effects. Variability was gauged using the I^2^ measure and Chi-square test; values of *p* < 0.1 or I^2^ > 50% were considered indicative of notable variability. All summary diagrams and figures were created with uniform styling (Arial 10-point type, consistent axis labels, symbols for weight relative to accuracy, and capitalized figure headings) to guarantee visual consistency.

## 3. Results

### 3.1. Summary of the Included Studies

**Figure 1:**
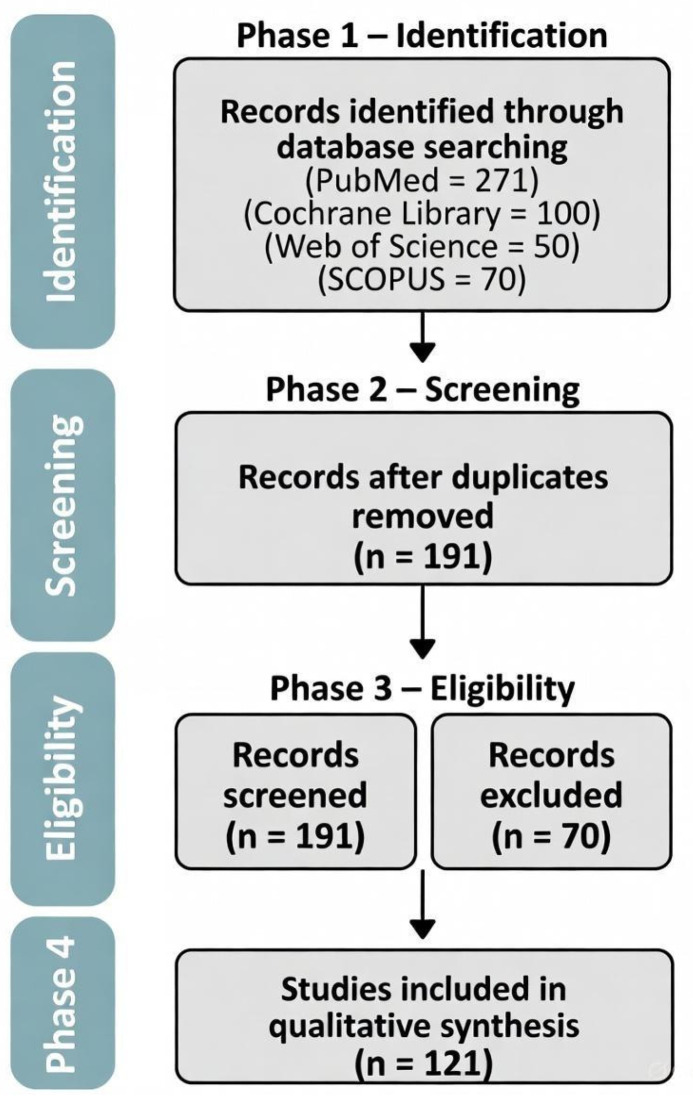
PRISMA detailed analysis of our literature search.

From 491 entries identified via database searches, 191 duplicates were deleted, leaving 300 distinct entries for title/summary examination. We selected nine investigations [4,5,6,17,18,19,20,21,22] for our examination: one randomized trial [19], one prospective matched study [17], and seven retrospective studies [4,5,6,18,20,21]. The thorough outcomes of our literature exploration are shown in the PRISMA diagram of Figure 1. All selected investigations compared the performance and reliability measures of LESS and CLS for the excision of non-malignant ovarian conditions. A total of 1368 females were selected in our examination: 702 females in the LESS category and 666 in the CLS category. The average age of the selected subjects was 30.6 ± 6.3. The average BMI in the LESS category was 25.7 ± 3.06, while the mean BMI in the CLS was 26.2 ± 3.1. The average ovarian condition size in cm was 7.2 ± 2.7 in the LESS category and 7.1 ± 2.2 in the CLS category. Table 1 and Table 2 present a thorough outline of the traits of the involved investigations and the population details of the females selected.

### 3.2. The Results of the Quality Assessment

**Figure 2:**
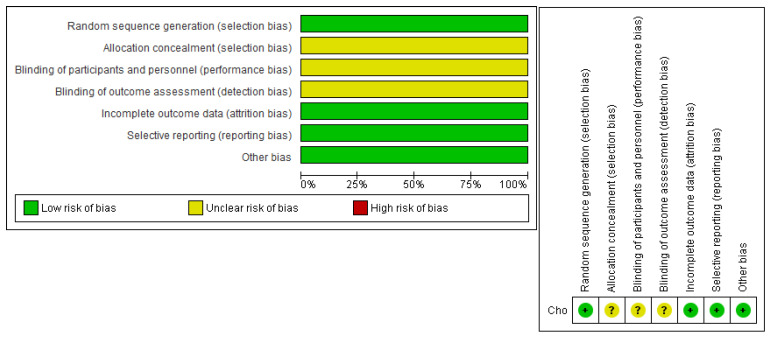
Detailed results of our quality assessment of the included studies.

The average rating of the non-randomized investigations was 10.7 out of a possible 14. Table 3 offers a thorough display of the quality assessment of the non-randomized investigations. Concerning the randomized controlled investigation [19], Cho et al. were determined to be at low risk in the randomization domain. However, the investigation did not provide sufficient details about blinding and choice bias, which then had to be rated as “uncertain.” Complete details of this are shown in Figure 2.

### 3.3. Analysis of Outcomes

1. Complete Operative Time (in Minutes)

**Figure 3:**
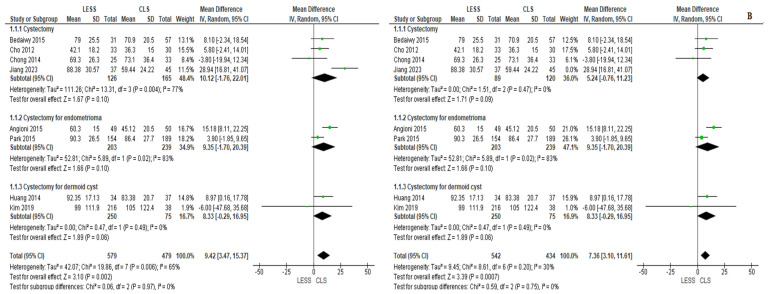
Meta-analysis of total operative time (in minutes).

Almost all the selected investigations reported the complete operative time for both methods. Our examination indicated that LESS was linked with a prolonged operative time than CLS (MD = 9.42 [3.47, 15.37], *p* = 0.002), with notable variability (I^2^ = 65%). We carried out a subgroup examination according to the kind of condition removed to obtain further understanding. The endometrioma subgroup indicated a prolonged operative time for LESS (MD = 9.35 [−1.70, 20.39], *p* = 0.10) (I^2^ = 83%). The dermoid cysts subgroup also indicated a prolonged operative time for LESS (MD = 8.33 [−0.29, 16.95], *p* = 0.06) with no variability (I^2^ = 0%). The final subgroup encompassed all remaining investigations and likewise showed a prolonged operative time for LESS (MD = 10.12 [−1.76, 22.01], *p* = 0.10) with considerable variability (I^2^ = 77%). In this subgroup, variability was effectively addressed by the removal of Jiang et al. [4], as displayed in Figure 3.

2. Approximated Blood Loss (EBL) (in mL)

**Figure 4:**
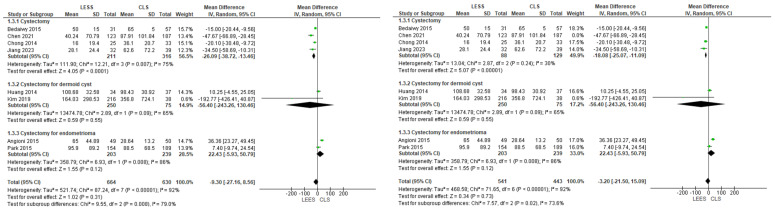
Meta-analysis of estimated blood loss.

Eight investigations reported that the operator approximated blood loss for this parameter. The CLS category had a greater EBL, but the difference was not statistically significant (MD = −9.30 [−27.16, 8.56], (*p* = 0.31)); I^2^ = 92%. We again carried out subgroup analyses based on condition type. In the dermoid cyst category, both categories reported comparable values of EBL (MD = −56.40 [−243.26, 130.46], (*p* = 0.55)); I^2^ = 65%. In the endometrioma category, there was no significant difference between the categories with greater values of EBL in the LESS (MD = 22.43 [−5.93, 50.79], (*p* = 0.12)); I^2^ = 86%. The final category (all others) supported the LESS category significantly with variable results (MD = −26.09 [−38.72, −13.46], (*p* = 0.001)); I^2^ = 75%, as displayed in Figure 4.

3. Complete Length of Hospital Time (in Days)

**Figure 5:**
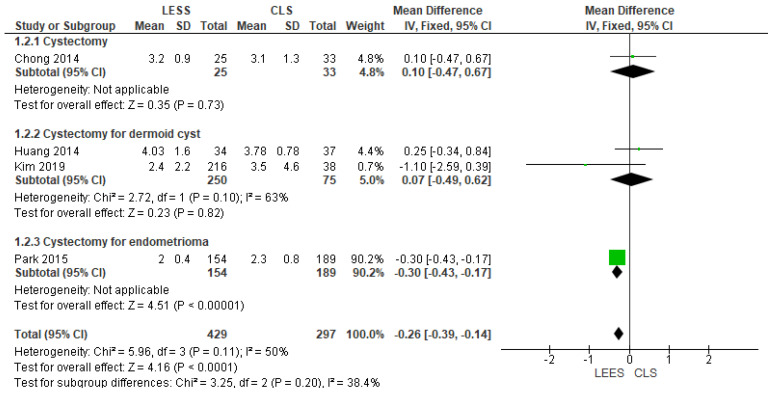
Meta-analysis of postoperative hospital stay (in days).

This parameter was reported by four investigations [6,20,21,22]. Our examination showed that the LESS category was linked with a briefer hospitalization time compared with CLS (MD = −0.26 [−0.39, −0.14], (*p* = 0.001). The combined examination was uniform (*p* = 0.11)); I^2^ = 50%, as displayed in Figure 5.

4. VAS Pain Scale Rating 24 h After Operation

**Figure 6:**
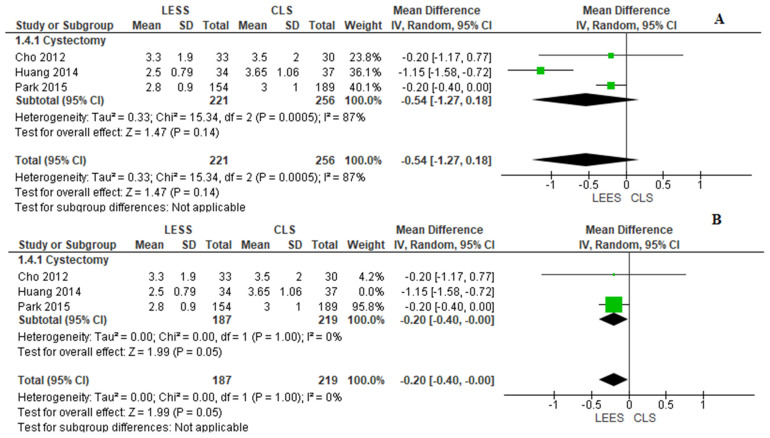
Meta-analysis of visual analog scale pain scores at 24 h after surgery.

This parameter was reported by three investigations [6,18,20]. In general, there was no significant difference between the two categories (MD = −0.54, 95% CI [−1.27, 0.18], *p* = 0.14) with substantial variability (I^2^ = 87%). Adjustment examination indicated that variability was mainly due to Huang et al. [21], which alone used an Octoport system and extracorporeal excision technique, methodological aspects not found in the other two investigations. When this unique investigation was removed post hoc, variability was removed (I^2^ = 0%), and the combined estimate indicated slightly lower 24-h discomfort ratings in the LESS category (MD = −0.20, 95% CI [−0.40, −0.00], *p* = 0.05), as displayed in Figure 6.

5. VAS Pain Scale Rating 6 h After Operation

**Figure 7:**
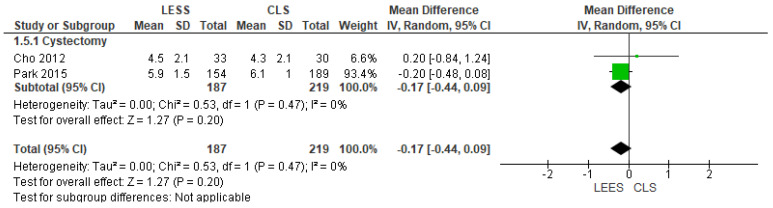
Meta-analysis of visual analog scale pain scores at 6 h after surgery.

Both methods were linked with comparable discomfort ratings six hours after operation (MD = −0.17 [−0.44, 0.09], (*p* = 0.20)). Our examination of the data was uniform (*p* = 0.47); I^2^ = 0%, as displayed in Figure 7.

6. Opioid Pain Relief Usage

**Figure 8:**
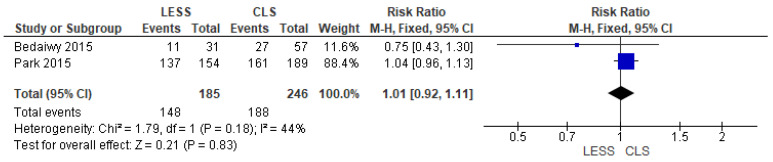
Meta-analysis of the rate of required narcotic analgesia in the postoperative period.

We gauged the frequency of needed pain relief usage after both methods. The frequency of needed analgesia in the LESS category was 148 in 185 instances, while the frequency in the CLS category was 188 in 246. The variance between both categories was not statistically meaningful (RR = 1.01 [0.92, 1.11], (*p* = 0.83). The combined examination was uniform (*p* = 0.18)); I^2^ = 44%, as displayed in Figure 8.

7. Change in Hemoglobin Level (HB) (in g/dL)

Five investigations (4,6,19,21,22) reported the variation in hemoglobin level after operation. Both categories were linked with similar reductions in HB with uniform examination (MD = 0.07 [−0.08, 0.22], (*p* = 0.36)).

8. Postoperative Complications

**Figure 9:**
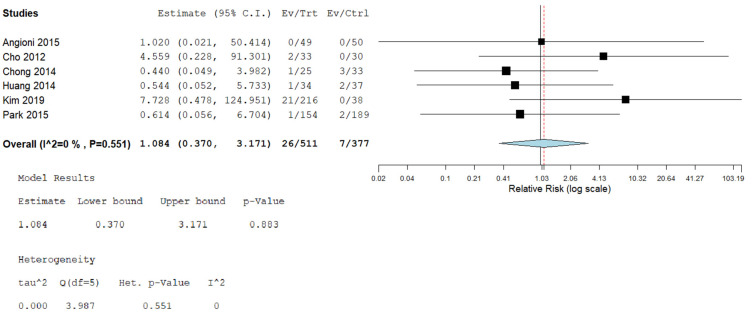
Meta-analysis of the rate of postoperative complications.

Six investigations assessed the reported postoperative issues of the methods. The frequency of postoperative issues was comparable between both categories (RR = 1.084 [0.370, 3.171], (*p* = 0.883). The combined examination was uniform (*p* = 0.5)); I^2^ = 0%, as displayed in Figure 9.

## 4. Discussion

Our findings revealed heterogeneity potentially arising from several sources: (i) differences in surgeon and facility expertise with single-port procedures, including the learning curve involved; (ii) variations in single-port equipment (such as commercial versus custom-built ports, and rigid versus flexible or jointed tools); (iii) employment of extracorporeal compared to fully intracorporeal excision methods; (iv) variations in cyst types (like endometrioma, dermoid, or other categories); and (v) site-specific practices in ovarian repair (suturing versus sealing products). Since most reports lacked patient-specific details on surgeon caseload, precise equipment used, or progress in skill acquisition, we could not perform aggregated regression to quantitatively investigate these aspects.

The overall extension of surgical duration by around 9.4 min remains minor and probably does not result in notable extra risks from sedation, while the 0.26-day decrease in inpatient duration—despite being limited—could nevertheless yield practical cost reductions and better individual outcomes in locations where standard practice includes overnight stays following endoscopic accessory procedures.

## 5. Comparison of Recent Literature

There have been two recent meta-analyses on this topic, although both were smaller in sample size than the present study. The most recent, by Lin et al. in 2020 [24], examined the postoperative complications and operative time for LESS and CLS in benign ovarian cysts. Similarly to our results, they found that LESS was associated with a longer operative time. The other recent meta-analysis, Schmitt et al. [25] in 2017, found no differences in operating time. This is likely due to their analysis including cases of salpingo-oophorectomy, where cauterization of the pedicles is largely used instead of laparoscopic suturing. Unilateral salpingo-oophorectomy is generally a faster procedure than ovarian cystectomy, which may have contributed to differences in operative time. Further evidence of this is that in two of our included studies, Bedaiwy et al. [5] and Park et al. [6], it was commented that there was little change in operative time when excluding the time required to suture the ovarian capsule. In the opinion of these authors, this accounted for the majority of the difference in operative time, likely due to the difficulty of laparoscopic suturing with a single port. These findings suggest that with increased surgical experience, careful selection of the surgical area, and the use of specialized equipment, the operative time for LESS may be reduced.

Our results also demonstrated that the patients who underwent LESS had a shorter hospital stay compared to those who underwent CLS, which may be the advantage of LESS in terms of recovery and general hospitalization. This finding is also consistent with several previous studies. Although in the US, adnexal surgery is almost universally outpatient, in many countries, the typical hospital stay after minimally invasive surgery for benign adnexal masses can be 1 to 2 days. Jiang et al. [4], in 2023, reported an average hospital stay of approximately 3 days, which they reported was a result of the unusual discharge policies of the hospital. The hospital allegedly only discharged its patients after all stitches were removed, pathological results were reported, and there was no discomfort. In this unusual circumstance, Park et al. [6] still reported that the patients in the LESS group had a postoperative stay of 48 h or less compared to the patients in the CLS group. Other studies on this topic, including Fagotti et al. [26] and Yim et al. [27], also noted reduced postoperative length of stay after LESS adnexal surgery compared to conventional laparoscopic adnexal surgery. This corroborates our findings.

Overall, the incidence of postoperative complications was similar between both groups, which implies comparable safety profiles. There have also been several studies that reported no statistically significant differences in the chance of perioperative hemoglobin levels between the LESS and CLS groups [4,19], and recent studies showing similar pain scores 24 h after surgery. This also agrees with our findings. Nevertheless, there was no difference in pain scores between the two groups at the end of the first postoperative day and six hours postoperatively. The need for further administration of opioid pain medication after surgery was also similar for both groups, suggesting similar short-term postoperative pain requirements. The analysis of the change in hemoglobin post-operation revealed no significant difference between LESS and CLS, suggesting comparable safety profiles regarding blood loss and recovery.

Similarly, in the recent meta-analyses, Lin et al. [24] did not see significant variations in pain ratings at 4 h after surgery, but did observe that the LESS group needed less analgesia. With our larger analysis, this finding was no longer significant. The findings highlighted in Schmitt’s meta-analysis of the two groups claimed that both had comparable pain scores [24]. Therefore, there is broad agreement among the recent meta-analyses on this topic, with essentially no unexplained confounding findings. We would have liked to have included outcomes related to patient satisfaction with cosmesis, but unfortunately, we were unable to find studies addressing this outcome. Future prospective trials should routinely incorporate validated patient-reported outcome measures and cosmetic satisfaction scales (e.g., Body Image Questionnaire, Patient Scar Assessment Questionnaire, or Manchester Scar Scale) to objectively quantify this potential advantage of LESS over CLS.

## 6. Limitations

This meta-analysis has several important limitations. First, only one small randomized controlled trial was included; the majority of evidence comes from retrospective observational studies, increasing the risk of selection, performance, and confounding biases. Significant inter-study heterogeneity persisted for several outcomes despite subgroup and sensitivity analyses. Surgeon experience with LESS, learning-curve effects, variations in single-port devices, and ancillary instrumentation were inconsistently reported and could not be quantitatively assessed. Sensitivity analysis limited to randomized studies was not performed because it would have left insufficient studies for meaningful pooled estimates of most outcomes. Finally, validated cosmetic outcomes, patient satisfaction, and cost-effectiveness data remain largely unreported in the current literature.

## 7. Conclusions

While LESS is associated with longer operative time, it reduces hospital stay without increasing complications or postoperative pain. These benefits are most likely to be realized in high-volume centers with extensive advanced laparoscopic expertise and in carefully selected patients (smaller cysts, limited adhesions). Future high-quality randomized trials with larger sample sizes, standardized instrumentation, and inclusion of validated cosmetic and patient-satisfaction scales are needed to confirm these findings and better define the patient populations that benefit the most from LESS.

## Figures and Tables

**Table 1:** Demographic and clinical characteristics of included participants.

Study ID	Country	Included Population	Intervention	Study Design	Sample Size LESS	Sample Size CLS	Age (Mean, SD/(IQR)) LESS	Age (Mean, SD/(IQR)) CLS	BMI (Mean, SD/(IQR)) LESS	BMI (Mean, SD/(IQR)) CLS
Angioni 2015 [17]	Italy	Patients aged 18–45 years; unilateral endometrioma greater than 35 mm; no previous gynecological surgery or hormonal therapy 3 months before surgery.	Cystectomy for unilateral endometrioma	Prospective case–control study	49	50	30.25 ± 5.10	30.53 ± 4.55	20.42 ± 2.30	20.19 ± 2.58
Bedaiwy 2015 [5]	USA	Patients undergoing ovarian cystectomy for benign disease.	Cystectomy	Retrospective cohort	31	57	32 ± 5	34 ± 5	27 ± 3	28 ± 4
Chen 2022 [18]	China	Patients who underwent ovarian cystectomy by CLS or LESS.	Cystectomy	Retrospective cohort study	123	187	30.36 ± 7.36	30.88 ± 6.25	21.44 ± 3.77	21.58 ± 3.22
Cho 2012 [19]	Korea	Patients aged between 18 and 45 years; premenopausal status; presence of a unilateral adnexal mass, the largest diameter of the unilateral adnexal mass ranging between 4 cm and 10 cm in imaging studies; and normal cancer antigen (CA)-125 levels.	Cyst enucleation of a unilateral benign adnexal mass	RCT	33	30	29.5 ± 6.2	31.1 ± 7.2	21.4 ± 3.2	22.5 ± 3.3
Chong 2015 [20]	South Korea	Patients underwent cystectomy for the treatment of large ovarian cysts.	Extracorporeal cystectomy	Retrospective cohort study	25	33	23.3 ± 8.3	22.3 ± 4.5	21.1 ± 3.1	22.5 ± 3
Huang 2014 [21]	Taiwan	Patients receiving cystectomy for ovarian dermoid cysts.	Ovarian dermoid cystectomy	Retrospective case–control study	34	37	34.59 ± 10.18	34.73 ± 9.68	21.29 ± 2.52	21.14 ± 2.61
Jiang 2023 [4]	China	Patients aged 18–50 years; BMI ≤ 35; no acute infection or serious chronic disease, and ovarian cysts ≤ 15 cm in diameter.	Cystectomy	Retrospective cohort study	37	45	31.05 ± 8.28	34.11 ± 7.32	21.93 ± 3.37	22.46 ± 3.10
Kim 2019 [22]	Korea	Patients who underwent surgery (cystectomy) for ovarian mature cystic teratoma.	Ovarian dermoid cystectomy	Retrospective cohort study	216	38	29.2 (8–49)	31.6 (10–47)	21.5 (14.3–38.4)	22.7 (17.2–31.5)
Park 2015 [6]	Korea	Patients who underwent laparoscopic surgery due to ovarian endometriomas.	Cystectomy of endometrioma	Retrospective study	154	189	35.3 ± 6.9	33.4 ± 7.1	21.74 ± 7.9	21.25 ± 6.3

SD—Standard deviation, IQR—Interquartile range, LESS—Laparoendoscopic single-site surgery, CLS—Conventional laparoscopic surgery.

**Table 2:** Summary of the lesion size and histology by included studies.

Study ID	Previous Abdominal Surgery LESS	Previous Abdominal Surgery CLS	Mass Size (cm) LESS	Mass Size (cm) CLS	Mature Cystic Teratoma LESS	Mature Cystic Teratoma CLS	Mucinous Cystadenoma LESS	Mucinous Cystadenoma CLS	Serous Cystadenoma LESS	Serous Cystadenoma CLS	Others LESS	Others CLS
Angioni 2015 [17]	NR	NR	7.60 ± 3.56	7 ± 2	NR	NR	NR	NR	NR	NR	NR	NR
Bedaiwy 2015 [5]	11 (35.4)	19 (33.3)	6.5 ± 2.1	7.2 ± 2.1	10 (32.3)	21 (37)	8 (15.7)	13 (22.9)	9 (29)	14 (24.7)	4 (13)	9 (15.9)
Chen 2022 [18]	NR	NR	NR	NR	53	45	NR	NR	NR	NR	41	91
Cho 2012 [19]	4 (12.1%)	5 (16.7%)	6.6 ± 1.6	7.0 ± 1.7	22 (66.7%)	19 (63.3%)	3 (9.1%)	2 (6.7%)	4 (12.1%)	5 (16.7%)	4 (12.1%)	4 (13.3%)
Chong 2015 [20]	NR	NR	11.4 ± 4.2	9.7 ± 2.3	18 (72)	25 (76)	5 (20)	4 (12)	1 (4)	4 (12)	1 (4)	0
Huang 2014 [21]	7 (20.6)	4 (10.8)	7.05 ± 2.37	6.39 ± 2.27	34 (100)	37 (100)	NR	NR	NR	NR	NR	NR
Jiang 2023 [4]	14 (37.8)	15 (33.3)	5.86 ± 3.24	5.91 ± 1.94	22 (59.5)	21 (46.7)	4 (10.8)	4 (8.9)	3 (8.1)	4 (8.9)	8 (22)	16 (35.6)
Kim 2019 [22]	21 (9.7)	6 (15.7)	7.2 (13.0–67.7)	7.51 (34.0–178.0)	NR	NR	NR	NR	NR	NR	NR	NR
Park 2015 [6]	12 (7.8)	9 (4.8)	5.7 ± 2.1	6.1 ± 2.1	NR	NR	NR	NR	NR	NR	NR	NR

NR—Not reported, LESS—Laparoendoscopic single-site surgery, CLS—Conventional laparoscopic surgery.

**Table 3:** Quality assessment for the included studies.

Question	Angioni 2015 [17]	Bedaiwy 2015 [5]	Chen 2022 [18]	Chong 2015 [20]	Huang 2014 [21]	Jiang 2023 [4]	Kim 2019 [22]	Park 2015 [6]
1. Was the research question or objective in this paper clearly stated?	1	1	1	1	1	1	1	1
2. Was the study population clearly specified and defined?	1	1	1	1	1	1	1	1
3. Was the participation rate of eligible people at least 50%?	1	1	1	1	0	1	1	1
4. Were all the subjects selected or recruited from the same or similar populations (including the same time period)? Were the inclusion and exclusion criteria for being in the study prespecified and applied uniformly to all participants?	0	0	1	1	1	1	1	1
5. Was a sample size justification, power description, or variance and effect estimates provided?	0	0	0	0	0	0	0	0
6. For the analysis in this paper, were the exposure (s) of interest measured before the outcome(s) being measured?	1	1	1	1	1	1	1	1
7. Was the timeframe sufficient so that one could reasonably expect to see an association between exposure and outcome if it existed?	1	1	1	1	1	1	1	1
8. For exposures that can vary in amount or level, did the study examine different levels of exposure as related to the outcome (e.g., categories of exposure, or exposure measured as a continuous variable)?	1	1	1	1	1	1	1	1
9. Were the exposure measures (independent variables) clearly defined, valid, reliable, and implemented consistently across all study participants?	1	1	1	1	1	1	1	1
10. Was the exposure(s) assessed more than once over time?	0	0	0	0	0	0	0	0
11. Were the outcome measures (dependent variables) clearly defined, valid, reliable, and implemented consistently across all study participants?	1	1	1	1	1	1	1	1
12. Were the outcome assessors blinded to the exposure status of participants?	*	*	*	*	*	*	*	*
13. Was the loss to follow-up after baseline 20% or less?	1	1	1	1	1	1	1	1
14. Were key potential confounding variables measured and adjusted statistically for their impact on the relationship between exposure(s) and outcome(s)?	1	1	0	1	1	1	1	1
Total score (out of 14)	10/14	10/14	10/14	11/14	10/14	11/14	11/14	11/14

Key: 1 = Yes, 0 = No, * = Not reported.

## Data Availability

The compiled dataset supporting this meta-analysis will be made available upon publication to any researcher making a reasonable request via email to requests@marchandinstitute.org.

## Supplementary Materials

The following supporting information can be downloaded at: https://www.mdpi.com/article/10.3390/jcm14248739/s1, Table S1: PRISMA 2020 Checklist [28].
